# Prophylactic and therapeutic remdesivir (GS-5734) treatment in the rhesus macaque model of MERS-CoV infection

**DOI:** 10.1073/pnas.1922083117

**Published:** 2020-02-13

**Authors:** Emmie de Wit, Friederike Feldmann, Jacqueline Cronin, Robert Jordan, Atsushi Okumura, Tina Thomas, Dana Scott, Tomas Cihlar, Heinz Feldmann

**Affiliations:** ^a^Laboratory of Virology, National Institute of Allergy and Infectious Diseases, NIH, Hamilton, MT 59840;; ^b^Rocky Mountain Veterinary Branch, National Institute of Allergy and Infectious Diseases, NIH, Hamilton, MT 59840;; ^c^Biology Department, Gilead Sciences, Foster City, CA 94404;; ^d^Center for Infection and Immunity, Mailman School of Public Health, Columbia University, New York, NY 10032

**Keywords:** MERS-CoV, antiviral, animal model, remdesivir, therapy

## Abstract

The continued emergence of Middle East Respiratory Syndrome (MERS) cases with a high case fatality rate stresses the need for the availability of effective antiviral treatments. Remdesivir (GS-5734) effectively inhibited MERS coronavirus (MERS-CoV) replication in vitro, and showed efficacy against Severe Acute Respiratory Syndrome (SARS)-CoV in a mouse model. Here, we tested the efficacy of prophylactic and therapeutic remdesivir treatment in a nonhuman primate model of MERS-CoV infection, the rhesus macaque. Prophylactic remdesivir treatment initiated 24 h prior to inoculation completely prevented MERS-CoV−induced clinical disease, strongly inhibited MERS-CoV replication in respiratory tissues, and prevented the formation of lung lesions. Therapeutic remdesivir treatment initiated 12 h postinoculation also provided a clear clinical benefit, with a reduction in clinical signs, reduced virus replication in the lungs, and decreased presence and severity of lung lesions. The data presented here support testing of the efficacy of remdesivir treatment in the context of a MERS clinical trial. It may also be considered for a wider range of coronaviruses, including the currently emerging novel coronavirus 2019-nCoV.

## Significance

Middle East Respiratory Syndrome, caused by the MERS coronavirus (MERS-CoV), continues to cause severe respiratory disease with a high case fatality rate. To date, potential antiviral treatments for MERS-CoV have shown limited efficacy in animal studies. Here, we tested the efficacy of the broad-acting antiviral remdesivir in the rhesus macaque model of MERS-CoV infection. Remdesivir reduced the severity of disease, virus replication, and damage to the lungs when administered either before or after animals were infected with MERS-CoV. Our data show that remdesivir is a promising antiviral treatment against MERS that could be considered for implementation in clinical trials. It may also have utility for related coronaviruses such as the novel coronavirus 2019-nCoV emerging from Wuhan, China.

Since its discovery in 2012, cases of Middle East Respiratory Syndrome coronavirus (MERS-CoV) have continued to emerge, with the current case count close to 2,500 cases, and a case fatality rate ∼35% (1). This continuous emergence of MERS-CoV infections in Saudi Arabia and its ability to spread through human-to-human transmission has prompted the World Health Organization to include MERS on their list of emerging diseases likely to cause major epidemics and for which countermeasures are urgently needed (2). Through the Coalition for Epidemic Preparedness Innovations, MERS-CoV vaccines are going to advance through preclinical and clinical trials (3), but, despite the urgent need, a similar initiative does not exist for the development and clinical testing of antivirals effective against MERS-CoV.

Remdesivir (GS-5734) is a nucleotide prodrug that has broad antiviral activity against viruses from different families in vitro (4), and therapeutic efficacy in nonhuman primate models of lethal Ebola virus and Nipah virus infection (5, 6). Studies in human airway epithelial cells showed that remdesivir also inhibits replication of a wide range of coronaviruses, including MERS-CoV (7). Efficacy studies in mice showed that remdesivir had therapeutic efficacy against Severe Acute Respiratory Syndrome (SARS)-CoV and MERS-CoV in Ces1c^−/−^ mice, deficient in a secreted carboxylesterase responsible for poor pharmacokinetics profile of remdesivir in mice, when administered before the peak of virus replication (7, 8). In vitro studies with mouse hepatitis virus showed that remdesivir inhibits coronavirus replication through interference with the viral polymerase, despite the presence of a viral proofreading exoribonuclease (9). Importantly, coronaviruses partially resistant to inhibition by remdesivir, obtained in vitro following >20 passages in the presence of GS-441524, a parent nucleoside that is metabolized into the same active triphosphate metabolite, were still sensitive to higher concentrations of remdesivir, and fitness was impaired in the resistant viruses as compared to wild-type MERS-CoV (9). With these promising data in mind, we tested the prophylactic and therapeutic efficacy of remdesivir treatment in a nonhuman primate model of MERS-CoV infection, the rhesus macaque (10).

## Results

### Remdesivir Reduces Clinical Signs in Rhesus Macaques upon Prophylactic and Therapeutic Treatment.

**Fig. 1.**
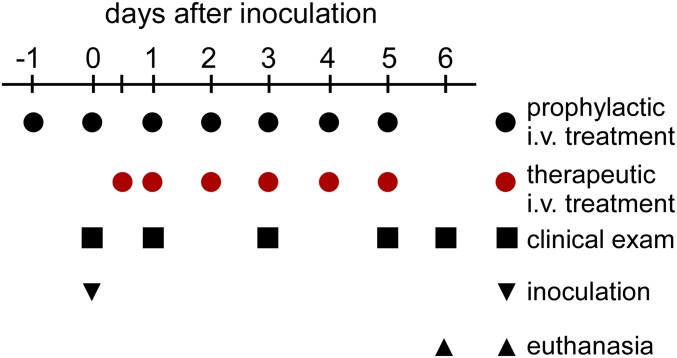
Study outline. To test the prophylactic and therapeutic efficacy of remdesivir treatment in the rhesus macaque model of MERS-CoV infection, three groups of six rhesus macaques were inoculated with MERS-CoV strain HCoV-EMC/2012; one group was administered 5 mg/kg remdesivir starting at 24 h before inoculation (black circles), and one group was administered 5 mg/kg remdesivir starting at 12 h after inoculation (red circles). One group of six control animals was i.v.-administered 1 mL/kg vehicle solution, with three animals receiving vehicle solution according to the prophylactic treatment schedule, and three animals receiving it according to the therapeutic treatment schedule. Treatment was continued once daily until 6 dpi, when all animals were euthanized. At 0, 1, 3, 5, and 6 dpi, clinical examinations were performed to monitor the health status of the animals.

To assess the efficacy of remdesivir to alleviate clinical signs of MERS-CoV infection, 18 rhesus macaques were randomly assigned to three groups of six animals. Three animals in the control group were treated with 1 mL/kg vehicle solution 24 h before MERS-CoV inoculation, and three animals were treated at 12 h post MERS-CoV inoculation. Another group of six rhesus macaques was treated prophylactically 24 h before MERS-CoV inoculation with 5 mg/kg remdesivir, and one group of six animals was treated therapeutically at 12 h postinoculation with MERS-CoV with 5 mg/kg remdesivir. Treatment was continued once daily until 6 d postinoculation (dpi), when animals were euthanized and necropsied (Fig. 1).

**Fig. 2.**
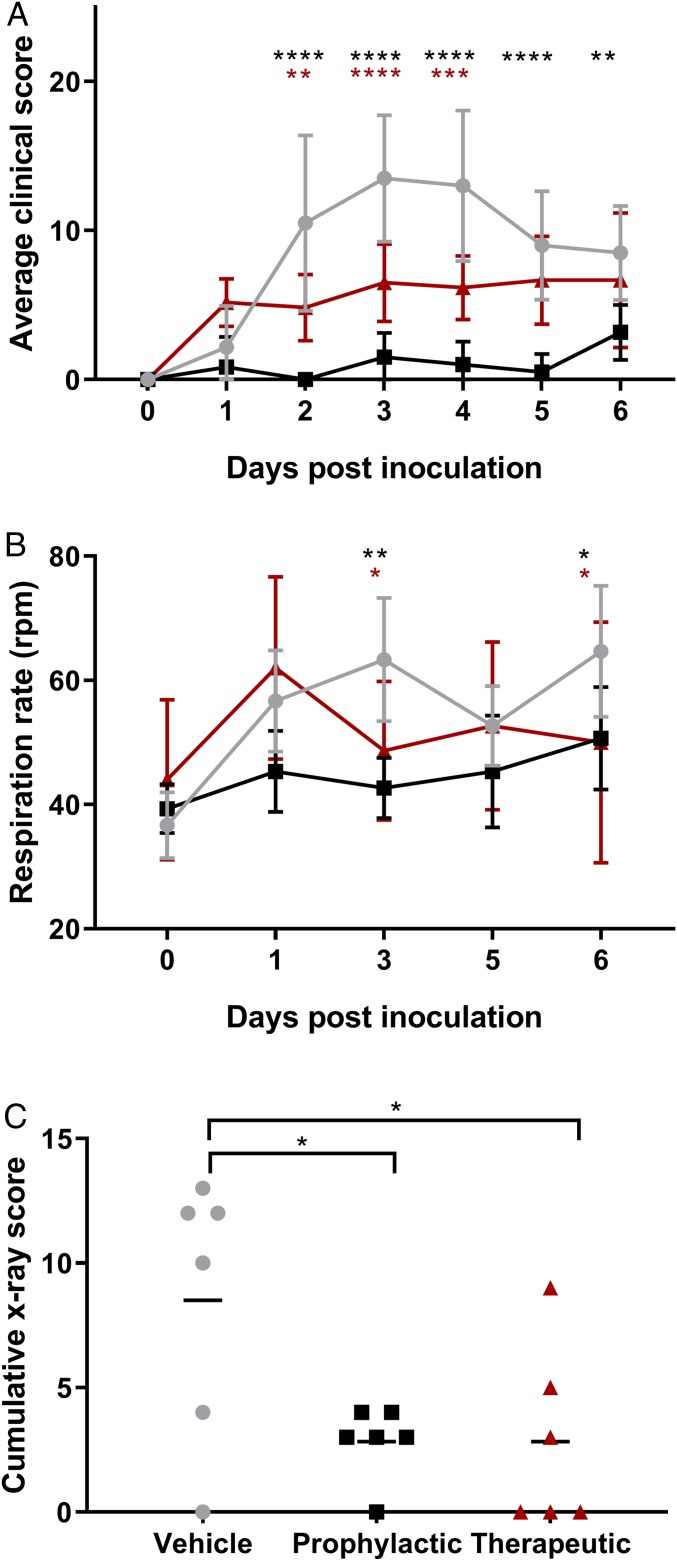
Clinical findings in rhesus macaques inoculated with MERS-CoV and treated with remdesivir. Three groups of six rhesus macaques were inoculated with MERS-CoV strain HCoV-EMC/2012; one group was i.v.-administered 1 mL/kg vehicle solution (vehicle control; gray circles), one group was administered 5 mg/kg remdesivir starting at 24 h before inoculation (prophylactic remdesivir; black squares), and one group was administered 5 mg/kg remdesivir starting at 12 h after inoculation (therapeutic remdesivir; red triangles). After inoculation, the animals were observed twice daily for clinical signs of disease and scored using a predetermined clinical scoring system (*A*). On 0, 1, 3, 5 and 6 dpi, clinical examinations were performed during which respiration rate was determined (*B*), and radiographs were taken. Radiographs were used to score individual lung lobes for severity of pulmonary infiltrates by a clinical veterinarian according to a standard scoring system (0: normal; 1: mild interstitial pulmonary infiltrates; 2: moderate pulmonary infiltrates perhaps with partial cardiac border effacement and small areas of pulmonary consolidation; 3: serious interstitial infiltrates, alveolar patterns and air bronchograms); the cumulative X-ray score is the sum of the scores of the six individual lung lobes per animal; scores shown are from 6 dpi (*C*). Asterisks indicate statistically significant difference in a two-way (*A* and *B*) or one-way (*C*) ANOVA with Dunnett’s multiple comparisons; black asterisks indicate statistical significance between the vehicle control and prophylactic remdesivir groups, and red asterisks indicate statistical significance between the vehicle control and therapeutic remdesivir groups. *P < 0.05; **P < 0.01; ***P < 0.001; ****P < 0.0001.

After inoculation with MERS-CoV on day 0, all animals were closely observed for signs of disease, and clinical scores were assigned according to a previously determined scoring sheet. All vehicle-treated animals displayed signs of disease, starting as early as 1 dpi, such as decreased appetite and ruffled fur; all vehicle-treated animals had respiratory signs such as increased respiration for 4 (*n* = 1) or 5 (*n* = 5) d after inoculation. The animals treated prophylactically with remdesivir did not show any respiratory signs of disease, but decreased appetite, possibly due to daily anesthesia, was noted in five of six animals. The animals treated therapeutically with remdesivir all displayed reduced appetites, and five out of six animals had increased respiration rates at 2 (*n* = 2), 3 (*n* = 2), or 4 (*n* = 1) d after inoculation. These observations are reflected in the clinical scores of the animals, with clinical scores in the prophylactically treated animals being statistically significantly lower than in vehicle-treated control animals at 2 to 6 dpi, and in the therapeutically treated animals at 2 to 4 dpi (Fig. 2*A*).

On days 0, 1, 3, 5, and 6, clinical examinations were performed on the animals, and respiration rates were determined on anesthetized animals. There was a clear increase in respiration rates in the vehicle-treated animals (Fig. 2*B*), while respiration rates in prophylactically treated animals remained normal throughout the study. Although respiration rate was increased in therapeutically treated animals at 1 dpi, respiration was statistically significantly lower than in vehicle-treated controls at 3 and 6 dpi (Fig. 2*B*). On examination days, radiographs were collected from all animals and analyzed for the presence of infiltrates; from 3 dpi onward, lung infiltrates became visible on X-ray (*SI Appendix*, Fig. S1). At 6 dpi, there was statistically significantly less infiltration in the lungs of animals treated both prophylactically and therapeutically with remdesivir as compared to vehicle-treated control animals (Fig. 2*C*).

### Reduced MERS-CoV Viral Lung Loads in Remdesivir-Treated Animals.

**Fig. 3.**
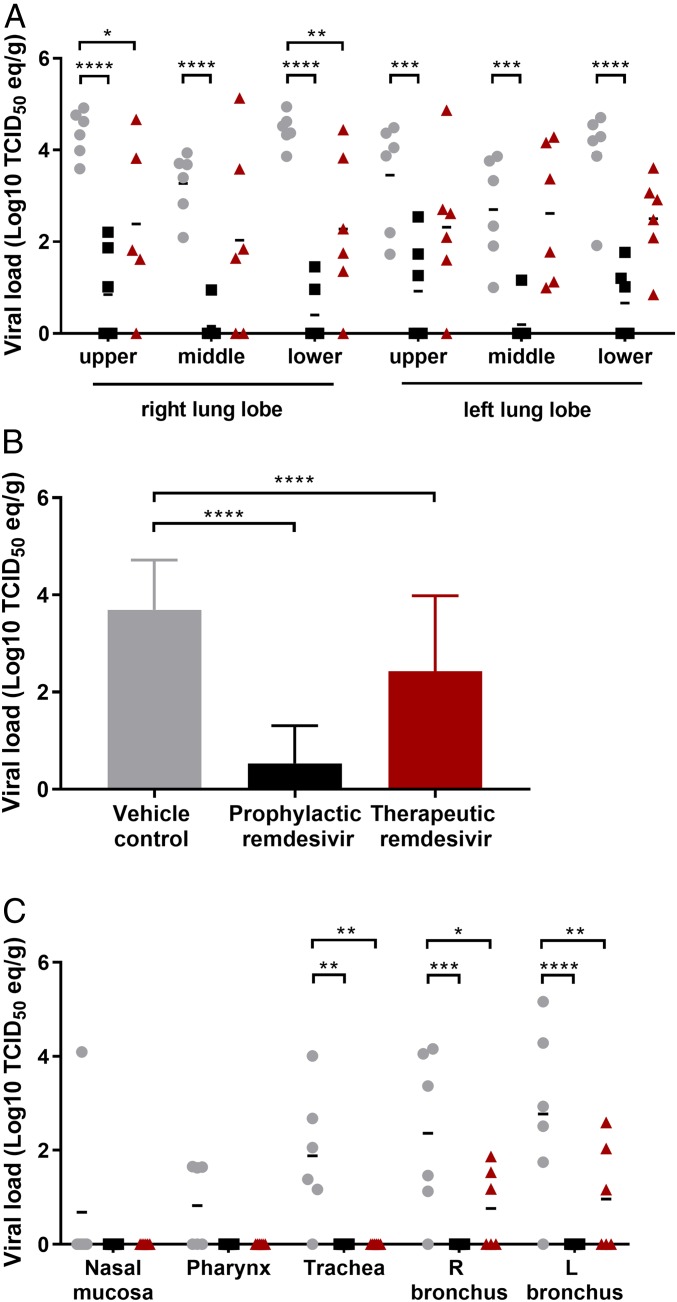
Viral loads in respiratory tract tissues of rhesus macaques inoculated with MERS-CoV and treated with remdesivir. Three groups of six rhesus macaques were inoculated with MERS-CoV strain HCoV-EMC/2012; one group was i.v.-administered 1 mL/kg vehicle solution (vehicle control; gray circles), one group was administered 5 mg/kg remdesivir starting at 24 h before inoculation (prophylactic remdesivir; black squares), and one group was administered 5 mg/kg remdesivir starting at 12 h after inoculation (therapeutic remdesivir; red triangles). Treatment was continued once daily until 6 dpi, when all animals were euthanized and necropsies were performed. At necropsy, tissue samples were collected from all six lung lobes, RNA was extracted, and viral load was determined as TCID50 equivalents per gram tissue. Individual animals and lung lobes are indicated (*A*), and averages and SDs per group (*B*). Similarly, viral loads were determined in additional tissues from the respiratory tract of each animal (*C*). R: right; L: left. Asterisks indicate statistically significant differences in a two-way ANOVA with Dunnett’s multiple comparisons. *P < 0.05; **P < 0.01; ***P < 0.001; ****P < 0.0001.

At 6 dpi, all animals were euthanized, and respiratory tissues were collected for quantitative analysis of the levels of viral RNA by qRT-PCR. Compared to vehicle-treated control animals, prophylactic remdesivir treatment resulted in significantly lower levels of MERS-CoV replication in the lungs, with lung viral loads 2.5 to 4 logs lower in each lung lobe (Fig. 3*A*). Although lung viral loads were, on average, lower in individual lung lobes after therapeutic treatment, this was statistically significant in only a few lung lobes, due to larger variation between animals in the therapeutically treated group (Fig. 3*A*). However, when all lung lobes were combined, the lung viral load in therapeutically treated animals was clearly lower than in vehicle-treated animals (Fig. 3*B*). Additionally, viral loads were significantly lower in trachea, bronchi, tonsils, and mediastinal lymph nodes of animals treated prophylactically and therapeutically with remdesivir than in vehicle-treated control animals (Fig. 3*C* and *SI Appendix*, Fig. S2); viral RNA was not detected in kidney tissue samples (*SI Appendix*, Fig. S2).

### Reduced Gross and Histologic Lung Lesions upon Remdesivir Treatment.

**Fig. 4.**
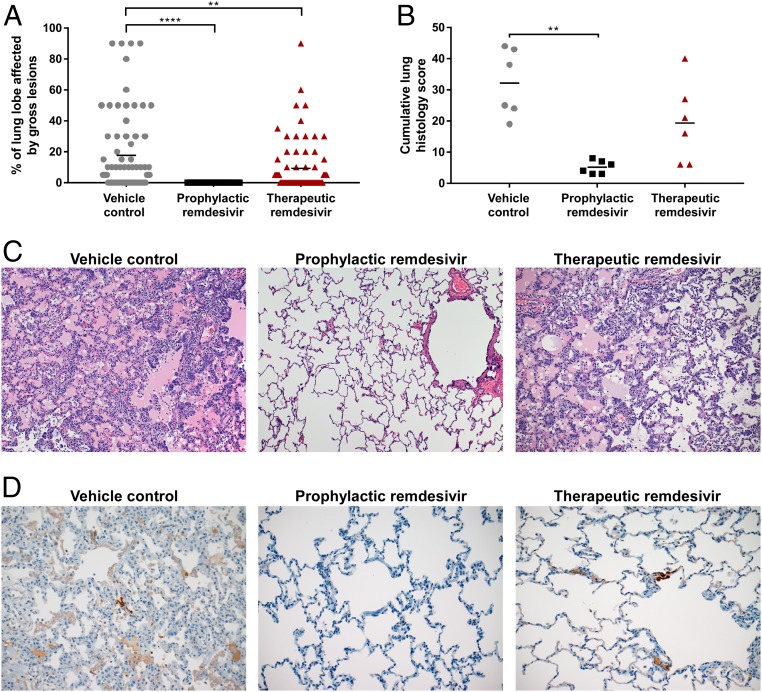
Pathological findings in the lungs of rhesus macaques inoculated with MERS-CoV and treated with remdesivir. Three groups of six rhesus macaques were inoculated with MERS-CoV strain HCoV-EMC/2012; one group was i.v.-administered 1 mL/kg vehicle solution (vehicle control; gray circles), one group was administered 5 mg/kg remdesivir starting at 24 h before inoculation (prophylactic remdesivir; black squares), and one group was administered 5 mg/kg remdesivir starting at 12 h after inoculation (therapeutic remdesivir; red triangles). Treatment was continued once daily until 6 dpi, when all animals were euthanized and necropsies were performed. At necropsy, the percentage of each lung lobe affected by gross lesions was estimated by a board-certified veterinary pathologist (*A*). Lung samples were collected and stained with H&E and analyzed for the presence of lesions by a board-certified veterinary pathologist. Each lung was given a score from 0 to 4 based on the abundance of lesions; the cumulative histology score is the sum of the scores of the six individual lung lobes per animal (*B*). One representative H&E image was chosen for each group (magnification: 100×) (*C*). Lung samples were also stained with a polyclonal α-MERS-CoV antibody; one representative image was chosen for each group (magnification: 200×) (*D*). Images in *C* and *D* were chosen as representative images of lung lesions and antigen expression, respectively, rather than being images from consecutive tissue slides. Asterisks indicate statistically significant differences in a two-way ANOVA with Dunnett’s multiple comparisons. **P < 0.01; ****P < 0.0001.

Upon necropsy, the area of each lung lobe affected by gross lesions was estimated by a board-certified veterinary pathologist. Gross lung lesions were present in several lung lobes of all of the vehicle-treated control animals (Fig. 4*A*). In contrast, gross lung lesions were completely absent in the lungs of animals that received prophylactic remdesivir treatment. In animals treated therapeutically with remdesivir, there were obvious gross lesions present in five out of six animals; however, the total area of lungs affected by gross lesions was statistically significantly smaller than in vehicle-treated control animals (Fig. 4*A*).

In addition, the severity of histologic lung lesions was assessed by assigning a score for each lung lobe. The resulting cumulative lung histology score was compared between treatment groups to assess differences in the severity of histologic lesions. Cumulative lung histology scores were significantly lower in animals treated prophylactically with remdesivir (Fig. 4*B*). The large variation between animals in the therapeutically treated group meant that the lower average histology score did not reach statistical significance (Fig. 4*B*).

Histologically, all of the vehicle-treated control animals developed some degree of pulmonary pathology when inoculated with MERS-CoV. Lesions were multifocal, frequently centered on terminal bronchioles, and consisted of minimal to marked, interstitial pneumonia, characterized by thickening of alveolar septae by edema fluid and fibrin and small to moderate numbers of macrophages and fewer neutrophils. Alveoli contained moderate numbers of pulmonary macrophages and neutrophils. In areas with moderate to marked changes, there was abundant alveolar edema and fibrin with multifocal formation of hyaline membranes, as well as abundant type II pneumocyte hyperplasia. Perivascular infiltrates of inflammatory cells multifocally within and adjacent to affected areas of the lung were also observed (Fig. 4*C*). In contrast, all animals treated prophylactically with remdesivir had essentially normal pulmonary tissue with no evidence of coronavirus infection (Fig. 4*C*). Animals treated with remdesivir therapeutically demonstrated various levels of severity of coronaviral pneumonia. In two out of six animals, no histologic evidence of pneumonia was detected. In three animals, multifocal, minimal to moderate interstitial pneumonia was observed like that described for the control animals; however, the lesions were less severe than in the controls and not as widely distributed throughout the lung lobes. Only one out of six animals had moderate interstitial pneumonia that was indistinguishable from the vehicle-treated control animals in severity and distribution.

Immunohistochemical analysis for the presence of MERS-CoV antigen showed small numbers of antigen-positive type I pneumocytes in all vehicle-treated control animals and in five out of six animals treated therapeutically with remdesivir; there was no difference in number or distribution of antigen-positive cells in animals where antigen was detected. MERS-CoV antigen could not be detected in any of the animals treated prophylactically with remdesivir (Fig. 4*D*).

## Discussion

Prophylactic remdesivir treatment prevented MERS-CoV−induced clinical disease and lung lesions in rhesus macaques inoculated with MERS-CoV, and strongly inhibited MERS-CoV replication in respiratory tissues. Since nosocomial transmission accounts for approximately one-third of MERS-CoV cases (11), prophylactic remdesivir treatment of patients, contacts of patients, and healthcare personnel with high-risk exposure to a diagnosed MERS patient and at high risk of developing severe MERS due to underlying conditions (12) could be considered. Therapeutic remdesivir treatment also provided a clear clinical benefit, with a reduction in clinical signs and virus replication, and the absence of lung lesions in two out of six remdesivir-treated animals and a reduction in lesion severity in three additional animals. Absence of histologic lung lesions, as seen in two out of the six animals with therapeutic remdesivir treatment, has so far rarely been observed in studies testing the efficacy of MERS-CoV antivirals in nonhuman primate models (13–16); it has only been shown once before in one out of three common marmosets treated with hyperimmune plasma at 6 h after inoculation (17). Thus, although it is hard to compare different studies due to the fact that different species were used and treatment was initiated at different time points after inoculation, remdesivir appears to be one of the most promising antiviral treatments tested in a nonhuman primate model to date.

Therapeutic remdesivir treatment was administered at 12 h after inoculation with MERS-CoV, and, although this may seem relatively early after inoculation, it is close to the peak of MERS-CoV replication in the rhesus macaque model (10). A drug that inhibits virus replication may be of little use once virus replication has reached its peak, as was shown in vitro (9). However, in a considerable number of severe cases of MERS, viral RNA and infectious virus can still be detected in respiratory tract samples several weeks after the onset of symptoms (18, 19), with this prolonged virus replication most likely due to the presence of underlying conditions such as diabetes mellitus (18). Likewise, an increase in virus replication over a longer period of time was observed in immunocompromised rhesus macaques (20). Thus, remdesivir treatment could not only be of benefit to patients diagnosed with MERS early after symptom onset but may also improve recovery in those patients with severe cases of MERS where prolonged virus replication occurs.

Human safety data are available for remdesivir. It has been used on a compassionate basis in several unique cases of Ebola virus disease (21, 22), as well as on a large scale in the ongoing Ebola virus outbreak in the Democratic Republic of Congo (23), with around 400 treated patients. In addition, its efficacy is currently being tested in a clinical trial in Ebola virus disease survivors with prolonged virus shedding (24, 25). Although the efficacy of remdesivir was lower in the Ebola virus trial than that of the different antibody treatments tested, survival was increased as compared to overall survival rate in this outbreak.

Taken together, the data presented here on the efficacy of remdesivir in prophylactic and therapeutic treatment regimens, the difficulty of coronaviruses to acquire resistance to remdesivir (9), and the availability of human safety data warrant testing of the efficacy of remdesivir treatment in the context of a MERS clinical trial. Our results, together with replication inhibition by remdesivir of a wide range of coronaviruses in vitro and in vivo (7), may further indicate utility of remdesivir against the novel coronavirus 2019-nCoV emerging from Wuhan, China (26).

## Materials and Methods

### Ethics and Biosafety Statement.

All animal experiments were approved by the Institutional Animal Care and Use Committee of Rocky Mountain Laboratories, NIH and carried out by certified staff in an Association for Assessment and Accreditation of Laboratory Animal Care International accredited facility, according to the institution’s guidelines for animal use, and followed the guidelines and basic principles in the United States Public Health Service Policy on Humane Care and Use of Laboratory Animals, and the Guide for the Care and Use of Laboratory Animals. Rhesus macaques were housed in adjacent individual primate cages allowing social interactions, in a climate-controlled room with a fixed light−dark cycle (12-h light/12-h dark). Animals were monitored at least twice daily throughout the experiment. Commercial monkey chow, treats, and fruit were provided twice daily by trained personnel. Water was available ad libitum. Environmental enrichment consisted of a variety of human interaction, commercial toys, videos, and music. The Institutional Biosafety Committee (IBC) approved work with infectious MERS-CoV strains under BSL3 conditions. Sample inactivation was performed according to IBC-approved standard operating procedures for removal of specimens from high containment.

### Study Design.

To evaluate the effect of remdesivir treatment on MERS-CoV disease outcome, we used the rhesus macaque model of MERS-CoV infection that results in transient lower respiratory tract disease (10). Rhesus macaques were chosen because of the requirement of daily anesthesia and intravenous (i.v.) injections that were perceived to be problematic in the alternative nonhuman primate model of MERS-CoV infection, the common marmoset (27), due to their small size. All animals were randomly assigned to groups and inoculated as described previously with a total dose of 7 × 10^6^ TCID50 of MERS-CoV strain HCoV-EMC/2012 via intranasal, oral, ocular (1 × 10^6^ TCID50 each), and intratracheal (4 × 10^6^ TCID50) routes (10). In the first experiment, the efficacy of prophylactic remdesivir treatment was tested in one group of six rhesus macaques (all males; female rhesus macaques were not available from the supplier at the time of this study) treated with 5 mg/kg remdesivir in vehicle solution (5 mg/mL 12% sulfobutylether-β-cyclodextrin in water and hydrochloric acid, pH3.5) and three control rhesus macaques (all males) who received the same volume (1 mL/kg) of vehicle solution. This 5 mg/kg dosing in rhesus macaques is roughly equivalent to the 100-mg daily dosing used in humans in the Ebola virus clinical trials. Treatment was initiated at 24 h before virus inoculation and continued once daily until 6 dpi. After observing good efficacy of remdesivir upon prophylactic treatment, a second experiment was performed to assess its therapeutic efficacy. One group of six rhesus macaques (all males) was treated with 5 mg/kg remdesivir, and three control rhesus macaques (all males) received the same volume of vehicle solution. Due to the acute nature of the MERS-CoV model in rhesus macaques, therapeutic treatment was initiated at 12 h after inoculation with MERS-CoV and continued once daily until 6 dpi. Treatment was delivered as a slow i.v. bolus injection (total dose delivered over ∼5 min) administered alternatingly in the left or right cephalic and saphenous veins. The animals were observed twice daily for clinical signs of disease, using a standardized scoring sheet as described previously (28); the same person, who was blinded to the group assignment of the animals, assessed the animals throughout the study. The predetermined endpoint for this experiment was 6 dpi. Clinical examinations were performed at 0, 1, 3, 5, and 6 dpi on anesthetized animals. On examination days, clinical parameters such as body weight and respiration rate were collected, as well as dorsal−ventral and lateral chest radiographs. Chest radiographs were analyzed by a board-certified clinical veterinarian blinded to the group assignment of the animals. After euthanasia at 6 dpi, necropsies were performed. The percentage of gross lung lesions were scored by a board-certified veterinary pathologist blinded to the group assignment of the animals, and samples of the following tissues were collected: conjunctiva, nasal mucosa, mandibular lymph node, tonsil, pharynx, trachea, all six lung lobes, mediastinal lymph node, liver, spleen, kidney, and bladder. Histopathological analysis of tissue slides was performed by a board-certified veterinary pathologist blinded to the group assignment of the animals.

### Virus and Cells.

HCoV-EMC/2012 (Vero passage 6) was kindly provided by the Department of Viroscience, Erasmus Medical Center, Rotterdam, The Netherlands, and propagated once in VeroE6 cells in Dulbecco’s modified Eagle’s medium (DMEM) (Sigma) supplemented with 2% fetal calf serum (FCS) (Logan), 1 mM l-glutamine (Lonza), 50 U/mL penicillin, and 50 μg/mL streptomycin (Gibco) (virus isolation medium). Next-generation sequencing of our MERS-CoV inoculum revealed that there was a deletion in ORF5 in a small percentage of sequences (∼10%). VeroE6 cells were maintained in DMEM supplemented with 10% FCS, 1 mM l-glutamine, 50 U/mL penicillin, and 50 μg/mL streptomycin.

### qPCR.

Tissues (30 mg) were homogenized in RLT buffer, and RNA was extracted using the RNeasy kit (Qiagen) according to the manufacturer’s instructions. For detection of viral RNA, 5 µL of RNA was used in a one-step real-time RT-PCR upE assay (29) using the Rotor-Gene probe kit (Qiagen) according to instructions of the manufacturer. In each run, standard dilutions of a titered virus stock were run in parallel, to calculate TCID50 equivalents in the samples.

### Histopathology and Immunohistochemistry.

Histopathology and immunohistochemistry were performed on rhesus macaque tissues. After fixation for 7 d in 10% neutral-buffered formalin and embedding in paraffin, tissue sections were stained with hematoxylin and eosin (H&E). To detect HCoV-EMC/2012 antigen, immunohistochemistry was performed using an in-house rabbit polyclonal antiserum against HCoV-EMC/2012 (1:1,000) as a primary antibody. Stained slides were analyzed by a board-certified veterinary pathologist blinded to the group assignment of the animals.

### Statistical Analysis.

Statistical analyses were performed using GraphPad Prism software version 7.04. For analysis, the three vehicle control animals from the first and second experiment were combined to form one group of six animals.

### Data Availability Statement.

All data discussed here will be made available to readers upon request.

## Supplementary Material

Supplementary File
