# Supplementary material for: Prophylactic and therapeutic remdesivir (GS-5734) treatment in the rhesus macaque model of MERS-CoV infection
Source: Proc Natl Acad Sci U S A. 2020 Feb 13;117(12):6771–6. doi: 10.1073/pnas.1922083117 (PMC7104368; doi:10.1073/pnas.1922083117)
Supplement: Supplementary File [file pnas.1922083117.sapp.pdf]

PNAS
www.pnas.org

Supplementary Information for

Prophylactic and therapeutic remdesivir (GS-5734) treatment in the rhesus macaque model of MERS-CoV infection

Emmie de Wit, Friederike Feldmann, Jacqueline Cronin, Robert Jordan, Atsushi Okumura, Tina Thomas, Dana Scott, Tomas Cihlar and Heinz Feldmann

Corresponding author: Emmie de Wit
Email: emmie.dewit@nih.gov

This PDF file includes:

Figure S1
Figure S2

www.pnas.org/cgi/doi/10.1073/pnas.1922083117

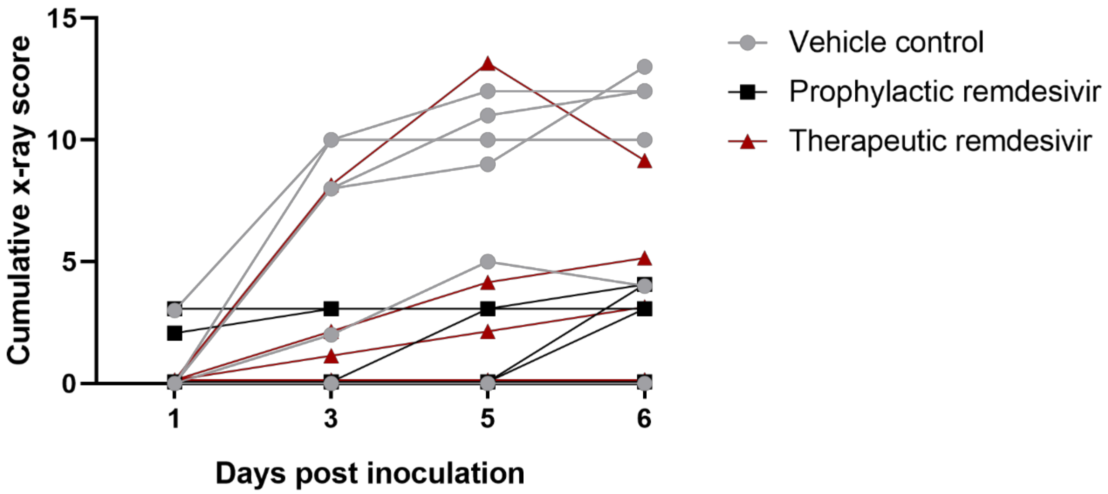

Fig. S1. Progression of pulmonary infiltrates over time in rhesus macaques inoculated with MERS-CoV and treated with remdesivir. Three groups of six rhesus macaques were inoculated with MERS-CoV strain HCoV-EMC/2012; one group was intravenously administered 1ml/kg vehicle solution (vehicle control, grey circles), one group was administered 5mg/kg remdesivir starting at 24 hrs before inoculation (prophylactic remdesivir, black squares), and one group was administered 5mg/kg remdesivir starting at 12 hrs after inoculation (therapeutic remdesivir, red triangles). On 0, 1, 3, 5 and 6 dpi radiographs were taken. Radiographs were used to score individual lung lobes for severity of pulmonary infiltrates by a clinical veterinarian according to a standard scoring system (0: normal; 1: mild interstitial pulmonary infiltrates; 2: moderate pulmonary infiltrates perhaps with partial cardiac border effacement and small areas of pulmonary consolidation; 3: serious interstitial infiltrates, alveolar patterns and air bronchograms); the cumulative x-ray score is the sum of the scores of the six individual lung lobes per animal.

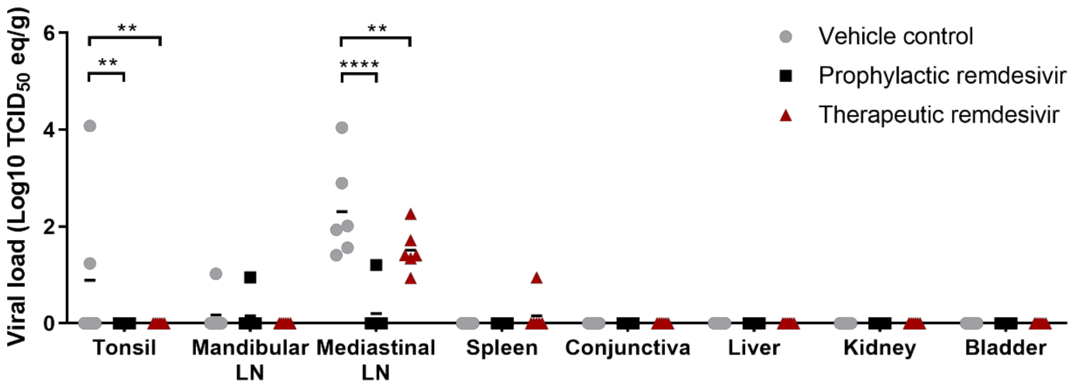

Fig. S2. Viral loads in extra-respiratory tissues of rhesus macaques inoculated with MERS-CoV and treated with remdesivir. Three groups of six rhesus macaques were inoculated with MERS-CoV strain HCoV-EMC/2012; one group was intravenously administered 1ml/kg vehicle solution (vehicle control, grey circles), one group was administered 5mg/kg remdesivir starting at 24 hrs before inoculation (prophylactic remdesivir, black squares), and one group was administered 5mg/kg remdesivir starting at 12 hrs after inoculation (therapeutic remdesivir, red triangles).). Treatment was continued once daily until 6 dpi when all animals were euthanized and necropsies were performed. At necropsy, tissue samples were collected, RNA was extracted and viral load determined as TCID50 equivalents per gram tissue. Individual animals and tissues are indicated. LN: lymph node. Asterisks indicate statistically significant differences in a 2-way ANOVA with Dunnett's multiple comparisons. ** P<0.01; **** P<0.0001.Fig. S2.
